# Medical net cost of low alcohol consumption - a cause to reconsider improved health as the link between alcohol and wage?

**DOI:** 10.1186/1478-7547-7-17

**Published:** 2009-10-23

**Authors:** Johan Jarl, Ulf G Gerdtham, Klara Hradilova Selin

**Affiliations:** 1 Health Economics & Management, Institute of Economic Research, Lund University, Lund, Sweden; 2 Center for Primary Health Care Research, Lund University/Region Skåne, Lund, Sweden; 3 Economics Department, Lund University, Lund, Sweden; 4 Social Research on Alcohol and Drugs (SoRAD), Stockholm University, Stockholm, Sweden

## Abstract

**Background:**

Studies have found a positive effect of low/moderate alcohol consumption on wages. This has often been explained by referring to epidemiological research showing that alcohol has protective effects on certain diseases, i.e., the health link is normally justified using selected epidemiological information. Few papers have tested this link between alcohol and health explicitly, including all diseases where alcohol has been shown to have either a protective or a detrimental effect.

**Aim:**

Based on the full epidemiological information, we study the effect of low alcohol consumption on health, in order to determine if it is reasonable to explain the positive effect of low consumption on wages using the epidemiological literature.

**Methods:**

We apply a non-econometrical cost-of-illness approach to calculate the medical care cost and episodes attributable to low alcohol consumption.

**Results:**

Low alcohol consumption carries a net cost for medical care and there is a net benefit only for the oldest age group (80+). Low alcohol consumption also causes more episodes in medical care then what is saved, although inpatient care for women and older men show savings.

**Conclusion:**

Using health as an explanation in the alcohol-wage literature appears invalid when applying the full epidemiological information instead of selected information.

## Introduction

Alcohol consumption has been shown in a number of studies to have a positive effect on wages, although the shape of the relationship differs between studies. For example, Peters [1], van Ours [2] and Zarkin et al. [3] find a positive effect of alcohol consumption, at least for men, while Lee [4] finds this effect only for moderate consumption. Hamilton & Hamilton [5], Barrett [6], Heien [7] and French & Zarkin [8] find a positive effect of moderate drinking but also a negative effect of heavy drinking. Several explanations have been suggested to explain the wage premium of low and moderate alcohol consumption such as family background and genetic endowment [4], social networking [2], and health [[2,6] and [9]]. With regard to health, the hypothesis is that alcohol has been shown to have a protective effect on some diseases, mainly coronary heart disease, which in turn is expected to increase the labour force/market productivity [6]. However, most studies ignore testing this link between alcohol and wage explicitly and do not discuss the fact that alcohol have been shown to have a detrimental effect on a number of other diseases.

Experiences from cost studies on alcohol, based on the literature of relative risks of alcohol consumption, seem to contradict health as a possible explanation of the alcohol-wage effect. The cost-of-alcohol studies performed in Australia [10] and Canada [11] include both beneficial and detrimental effects of alcohol consumption and thus produce a net effect of alcohol consumption, measured in monetary values. Both studies show a net societal loss for health care resulting from alcohol consumption. This indicates that alcohol reduces health overall and should therefore be expected to have a negative effect on wage. However it may be argued that the results from Australia and Canada are driven by heavy consumption and that consuming low and moderate amounts are indeed beneficial to health. Looking at the epidemiological literature on which the cost-of-illness (COI) studies are based, alcohol consumption is deemed to increase the risk for many more diseases than for which it is deemed to decrease the risk, even when looking at low and moderate consumption. This seems counterintuitive to the health link made in the alcohol-wage literature. However, the sheer number of diseases is for obvious reasons not of interest but rather the net health effect of these diseases, i.e. prevalence and severity. Establishing the net effect of alcohol-related diseases from low alcohol consumption on health in relation to abstainers can help explain the issue. Three results of low alcohol consumption are possible: 1) a net beneficial effect, 2) no significant effect, or 3) a net detrimental effect. The third result, and to a lesser degree the second, would indicate a contradiction between the COI-literature and the health link in the alcohol-wage literature which in turn would require further analysis to reconcile the two separate areas of research.

Econometric methods are often used to estimate issues such as the effect of alcohol consumption on health. However, these methods are not without problems, for example it is often suspected that alcohol consumption is endogenous. The endogeneity may arise from unobserved factors, such as preferences, that affect both alcohol and health, or may be caused by the reverse effect of health on alcohol. For example, if individuals with extra concern for their health simultaneously adjust their alcohol consumption to low levels and consume high levels of health care or if unhealthy individuals tend to use alcohol to self-medicate. One potential remedy of this problem is to use instrumental variable techniques, though finding instruments for alcohol is a non-trivial exercise. The present paper applies the COI approach to study whether low alcohol consumption has a net beneficial effect on health. Our justification for using this approach is not really avoidance of endogeneity problems but rather that COI studies are based on the epidemiological literature - the same literature as the alcohol-wage literature refers to when justifying the health link. It is therefore of great general interest to apply the COI method unconditionally by taking full account of all existing epidemiological data thereby reducing the risk that the health argument is drawn from selected results that fit with a priori expectations. It should be noted that endogeneity is expected to be a minor problem in the current study as only alcohol related diseases are included and as the epidemiological methods used (calculation of AAF, see below) have the purpose of separating the effect of alcohol consumption from other health related behaviours that have been shown to affect the same disease.

The issue under investigation in this study is the health effect of low alcohol consumption mediated through alcohol related diseases. This is measured by estimating the medical care costs and episodes caused by low alcohol consumption. We would argue that these two measures are appropriate proxies for catching the health effect, where the former should capture the severity of disease and the latter the actual number of cases. It is not obvious if medical care cost are at the risk of causing bias to the estimations and, if so, in what direction. An alternative approach for future research, that should complement the current study, would be to measure the actual health change following different alcohol related diseases, adjusted for age and gender.

## Methods

We use the COI approach to study whether low alcohol consumption has a net beneficial effect on health, measured as medical care costs and episodes in Sweden for the year 2002. We study the year 2002 for practical reasons as all data were identified and collected within the Swedish cost of alcohol project [12,13]. By converting the effect of each alcohol related disease into a common unit, i.e. money, the comparison of beneficial and detrimental health effects is facilitated. The net cost of alcohol consumption is not necessarily sufficient to determine the health effect since diseases can be more or less expensive to treat irrespective of actual health status. Therefore we also calculate and present the numbers of care episodes which, together with the cost, are deemed to give a good indication on the overall health effect of low alcohol consumption. As stated above, our main motivation for using this approach is to be able to apply the same information as the alcohol-wage literature implicitly refers to when justifying the health link, i.e. the epidemiological literature. However, this paper, in contrast to the alcohol-wage literature, takes full account of the information available in the epidemiological literature and thus considers the net effect of all alcohol-related diseases.

The COI approach has received much criticism over the years. Many issues, such as handling of mortalities and productivity costs, do not apply to this study as it only focuses on medical care costs. The calculation of medical care costs/episodes are straight forward and attract little problems, although for example the issue of calculating of attributable fractions remains (see below). We are following the methodological recommendations made in *International guidelines for estimating the costs of substance abuse *[14]. Methods in this field have been extensively developed and improved over the last decades and gained a position as useful tools in practical research.

The definition of low consumption used in this paper follows recommendations [15] and constitutes of 0-19.99 grams for women and 0-39.99 g for men, measured in pure alcohol per day. It could be questioned if someone consuming 39 g pure alcohol per day (19 g for women) should be considered a low consumer, and the results of the current study are expected to change if another (lower) cut-off point for low consumption are used. However, this is the conventional definition of low alcohol consumption on which also much of the epidemiological literature is based. The current definition of low alcohol consumption is considered the most appropriate among the alternatives. However, it should be noted that low alcohol consumption does not denote "safe" consumption, as the results below will make clear. Four consumption groups are used in total; abstainers, low consumption >0-<20 g pure alcohol per day for women and >0-<40 g for men, hazardous consumption >20-<40 g for women and >40-<60 g for men, and harmful consumption >40 g for women and >60 g for men. This definition of alcohol content is also considered better, due to its objective characteristic, than the vague 'standard drink' measure that differs between countries. A standard drink in Australia is for example 10 grams of pure alcohol while it is 6 g in Austria, 8 g in UK, 14 g in USA, and 19.75 g in Japan [16]. The division of the Swedish population into the different consumption groups is made using the Monitoring study for the years 2002 and 2004. The survey is a monthly telephone interview of 1'500 individuals aged 16-80 each month regarding different kinds of alcohol purchase in the last 30 days [17].

### Alcohol-related diseases

The only diseases included in this study are diseases that have been proven to be affected by alcohol consumption, see Table 1. These diseases and their associated relative risks, based on meta-analyses of individual studies, are adopted from Rehm et al. [11], with the exception of the relative risks for ischemic and haemorrhagic stroke that are taken from a reanalysis by Rehm (pers. com. 2005-10-07). Relative risks for mortality and morbidity are generally assumed to be the same for chronic alcohol-related diseases, although this can be debated [12]. For a disease to be included, the usual epidemiological criteria were applied (i.e. consistency, effect size, temporality and biological mechanisms). Injuries and chronic diseases fully attributable to alcohol consumption (e.g. alcoholic psychoses, alcohol abuse, and alcoholic gastritis) do not have associated relative risks and can therefore not easily be divided into different consumption groups. Two assumptions were therefore made regarding the cost division: 1) chronic diagnoses without relative risks were assumed to only affect the highest consumption group, i.e. harmful consumption, and 2) injuries were assumed to affect harmful and hazardous consumption in equal proportions. These assumptions affect only diseases with detrimental effects of alcohol consumption and therefore underestimate the roll of low consumption in the total burden of alcohol consumption which should be considered when interpreting the results. Included diseases with gender specific relative risks are shown in Table 1.

**Table 1:** Included alcohol related diseases with associated relative risks

**Condition**	**ICD-10**	**Relative risks**					
		**Low**		**Hazardous**		**Harmful**	
		**Wom**.	**Men**	**Wom**.	**Men**	**Wom**.	**Men**

*Malignant neoplasms*							
Mouth and oropharynx cancers	C00-C14	1.45	1.45	1.85	1.85	5.39	5.39
Stomach cancer	C16	1.07	1.07	1.15	1.15	1.32	1.32
Oesophageal cancer	C15	1.80	1.80	2.38	2.38	4.36	4.36
Liver cancer	C22	1.45	1.45	3.03	3.03	3.60	3.60
Laryngeal cancer	C32	1.83	1.83	3.90	3.90	4.93	4.93
Breast cancer	C50	1.14	Na	1.41	na	1.59	Na
Other neoplasms	D00-D48	1.10	1.10	1.30	1.30	1.70	1.70

*Diabetes*							
Diabetes mellitus	E10-E14	0.92	0.99	0.87	0.57	1.13	0.73

*Neuro-psychiatric conditions*							
Epilepsy	G40-G41	1.34	1.23	7.22	7.52	7.52	6.83

*Cardiovascular diseases*							
Hypertensive disease	I10-I15	1.40	1.40	2.00	2.00	4.10	4.10
Ischemic heart disease	I20-I24, I25.1-I25.9	0.82	0.82	0.83	0.83	1.00	1.12
Cardiac arrhythmias	I47-I49	1.51	1.51	2.23	2.23	2.23	2.23

*Cerebrovascular disease*							
Haemorrhagic stroke	I60-I62	0.74	1.12	1.04	1.40	1.94	1.54
Ischemic stroke	I63-I66	0.66	0.94	0.84	1.13	1.53	1.19
Oesophageal varices	I85	1.26	1.26	9.54	9.54	9.54	9.54

*Digestive diseases*							
Cirrhosis of the liver	K70, K74	1.30	1.30	9.50	9.50	13.00	13.0
Cholelithiasis	K80	0.82	0.82	0.68	0.68	0.50	0.50
Acute and chronic pancreatitis	K85, K86.1	1.3	1.3	1.8	1.8	3.2	3.2

*Skin diseases*							
Psoriasis	L40	1.58	1.58	1.60	1.60	2.20	2.20

Source: Rehm et al. (2006) [11].

Low consumption: women >0-<20 grams of pure alcohol per day; men >0-<40 g/day.

Hazardous consumption: women 20-<40 g/day; men 40-<60 g/day

Harmful consumption: women >40 g/day; men >60 g/day

### Alcohol-related care episodes

In order to establish the role of alcohol consumption for each alcohol-related disease, an alcohol attributable fraction (AAF) is calculated using the following well-known formula:



where no consumption is the counterfactual scenario and *i *denotes consumption categories, P_*i *_is the prevalence rate of consumption and RR_i _is the relative risk of the *i*:th category [11,18].

Applying the AAF to the total number of care episodes for a certain disease will produce the number of episodes that can be causally attributed to alcohol consumption [14]. As the above formula is constructed for calculation of total alcohol-related burden, a modified formula has been applied in order to calculate an AAF for each consumption group:



Using the modified formula normally results in an increased number of alcohol-related episodes, compared to the original formula. However, since there is no obvious reason to prefer one in front of the other, an additional adjustment is made. We calculate the *relative proportions *between consumption groups using the modified formula. We then apply this relative proportion to the total number of alcohol-related episodes, calculated using the original formula, for each diagnosis. Thus, a conservative number of alcohol-related episodes from low alcohol consumption are estimated. Examples of the calculations for two diseases are shown in Table 2.

**Table 2:** Example of calculations of number of care episodes and costs attributable to low alcohol consumption.

**Age group**	**Total number of inpatient cases**	**Alcohol attributable fraction**	**Total number of alcohol-related cases**^1^	**Disease specific cost**	**Total cost (millions)**^1^	**Attributable proportion**^2^	**Alcohol-related care episodes**^2^	**Alcohol-related costs (millions)**^2^
**Ischemic heart disease, men**								

18-29	31	-0.17	-5.4	39 861.06	-0.22	0.98	-5.34	-0.21
30-49	2 840	-0.19	-530.1	39 861.06	-21.13	0.99	-524.19	-20.89
50-64	15 109	-0.19	-2 805.1	39 861.06	-111.81	0.99	-2 763.37	-110.15
65-79	21 764	-0.16	-3 523.2	39 861.06	-140.44	1.00	-3 509.52	-139.89
80+	10 502	-0.14	-1 469.3	39 861.06	-58.57	1.00	-1 463.04	-58.32

**Epilepsy, women**								

18-29	360	0.46	165.2	23 629.21	3.90	0.34	56.15	1.33
30-49	726	0.41	299.9	23 629.21	7.09	0.40	119.98	2.84
50-64	767	0.39	299.9	23 629.21	7.09	0.43	128.05	3.03
65-79	787	0.24	192.2	23 629.21	4.54	0.67	128.67	3.04
80+	532	0.20	103.7	23 629.21	2.45	0.74	77.13	1.82

Prevalence data are taken from Johansson et al. (2006) [12]. Rows may not sum due to rounding. Monetary values in Swedish kronor, 2002.

^1^All consumption groups

^2^Low consumption

### Data

Primary care is the first instance of care in the Swedish health care system where basic medical investigation and treatment are given along with preventive care and rehabilitation. Outpatient care is specialist care, normally performed within hospitals but where the patient is not admitted nor stays over night, this also includes home care. For neither of these are national level data for visits available in Sweden that also includes diagnoses. We have therefore employed data from Västra Götalandsregionen, an administrative area in western Sweden, where a project for coding outpatient and primary care according to ICD-10 has been running for several years. The projects coverage rate has been increasing for each year, although still rather low for 2002, why data for year 2003 is used for primary care. The coverage rate in the used dataset is 26.9% for primary care and 45.0% for outpatient care. It is assumed that the disease distribution is the same for patients with and without an ICD-10 code, an assumption considered to be acceptable for alcohol related diseases that are not fully attributable to alcohol consumption, see below. Västra Götalandsregionen is considered a representative area of Sweden for statistical purposes [19]. Data for inpatient episodes are adopted from the Swedish National Inpatient Discharge Register with a coverage rate of 99% regarding main diagnosis. The register is deemed to be of very high quality and no adjustments are made.

Cost information for outpatient and primary care are gathered on a national level from Landstingsförbundet [20]. We estimate a standard cost per episode (total cost divided by total number of visits), adjusted for type of visit (different resource use and medical personnel). Inpatient care costs are calculated as a disease specific cost per episode. The actual cost of each episode is unknown but "shadow prices" are determined using an administrative process based on DRG-codes. Every DRG-code has a certain weight that is put in relation to the ward/hospital's total cost, giving the average disease-specific cost per episode which is used in this study. The cost is attributed for each discharge, so if a patient is admitted for the same disease several times over the year, the disease-specific cost is counted for each of these episodes. However, as the disease-specific cost is calculated from the ward/hospital's total cost for one year, the average disease-specific cost will be lower if patients are re-admitted due to premature discharges. The cost information is taken from two different health care administrative areas (Region Skåne and Stockholm County Council (SLL)). The assumption that Region Skåne and SLL are representative areas for Sweden as a whole could be questioned as these areas have a more urban characteristic, although it is not obvious how this would affect the results.

## Results

The net effect of low alcohol consumption is shown below. Five of the included 19 alcohol-related disease categories show protective effects for certain consumption groups and sexes (see Table 1). It is however important to take into account the prevalence of the different diseases as a very large increase in relative risk from consumption for a very rare disease will result in fewer alcohol attributable episodes and costs than a smaller increase in risk for a common disease. In addition, the disease specific cost of treatment in inpatient care is also important as differences are expected due to different length of stay and intensity of care between diseases. All figures are presented as net costs or care episodes, meaning that a negative number implies a protective effect of low alcohol consumption.

### Costs

The net medical cost resulting from low alcohol consumption totals to 187.3 million SEK (1 US$ in 2002 equals 9.35 SEK) out of which men's consumption stands for 64%, see Table 3. The highest net costs occur for the age group 50-64 followed by 30-49. The only age group, for both genders, that show a protective effect of low alcohol consumption on medical care costs are 80+. Dividing the net costs on the different care units show that inpatient care benefits from protective effects while outpatient- and primary care result in net costs. All age groups in inpatient care for women show a net protective effect of low alcohol consumption while this is true for age group 50-64 and above for men.

**Table 3:** Alcohol-related cost of medical care attributable to low consuming adults, Sweden 2002 (millions SEK)

	**Men**				**Women**			
	**Inpatient**	**Outpatient**	**Primary**	**Total**	**Inpatient**	**Outpatient**	**Primary**	**Total**
**18-29**	3.52	5.88	3.41	**12.82**	-3.86	8.19	5.09	**9.42**
**30-49**	1.18	19.04	21.73	**41.95**	-4.86	26.36	25.22	**46.72**
**50-64**	-38.03	24.81	58.65	**45.44**	-3379	29.79	62.72	**58.72**
**65-79**	-64.30	24.66	64.75	**25.10**	-92.71	25.06	79.02	**11.37**
**80+**	-35.98	7.61	22.84	**-5.54**	-112.82	10.46	43.70	**-58.66**

**All**	-133.60	82.00	171.37	**119.78**	-248.05	99.86	215.75	**67.57**

### Episodes of care

Given that the relationship between health care cost and actual health status could be questioned, we also calculate the number of care episodes related to low alcohol consumption in the Swedish health care sector during 2002 (Table 4). There is a net saving of care episodes in inpatient care for both genders. For men, this is a result from protective effect for individuals above 50 years of age, while the protective effect is valid for all age groups for women. Low alcohol consumption results in a rather large net increase of care episodes in outpatient- and primary care for both genders and all age groups, especially 50-79 year olds.

**Table 4:** Number of alcohol-related care episodes attributable to low consuming adults, Sweden 2002

	**Men**			**Women**		
	**Inpatient**	**Outpatient**	**Primary**	**Inpatient**	**Outpatient**	**Primary**
**18-29**	129	2 756	2 296	-112	3 836	3 426
**30-49**	233	8 916	14 626	-8	12 345	16 974
**50-64**	-239	11 620	39 478	-232	13 951	42 214
**65-79**	-693	11 548	43 578	-898	11 737	53 189
**80+**	-567	3 563	15 370	-1 751	4 898	29 416

**All**	-1 137	38 403	115 348	-3 000	46 767	145 218

## Discussion

Information from the epidemiological literature shows that the number of diseases where low alcohol consumption has a detrimental effect far exceeds the number of diseases with a protective effect. In addition does this study indicate that there is a net detrimental effect of low alcohol consumption, especially for working-aged individuals, measured as medical cost and number of care episodes. It thus seems that the health-link argument in the alcohol-wage literature fails to account for those studies where alcohol consumption is shown to increase the risk of disease. It is therefore doubtful if the common explanation of health as the link between alcohol consumption and increased wages is valid in its existing form. There is a beneficial effect of low alcohol consumption on medical cost for the age group 80 years and above, most likely following the increase in prevalence with older age in those diseases with a beneficial effect of alcohol. However, this age group is normally outside the workforce and thus receives no wages. The net detrimental effect of low alcohol consumption on working aged individuals is expected to have a negative effect on wages which is contradictory to the hypothesised health link in the alcohol-wage literature. We see no obvious reasons why the results of this study could not be applied to other (comparable) countries. On the contrary, as Sweden has a high prevalence of coronary heart disease as a fraction of total disease burden compared to other countries [21], we expect that the net cost and care episodes due to low alcohol consumption will be lower in Sweden than other countries. That is, since the study still shows non-positive effects on health care cost and episodes, this indicates that the results and conclusions can be applied to other countries as well. A note for concern might be the rather hazardous drinking pattern in Sweden [15]. However, the use of relative risks from international studies, due to lack of country specific information, and given that the hazardous pattern should reduce the beneficial effects, this should not hinder the application of the results from this study to other comparable countries.

Earlier work studying the effect of alcohol consumption on health care utilisation (compared to the alcohol-related health care utilisation in this study) have normally shown that alcohol use decreases utilisation compared to abstainers but also a negative relationship between different levels of consumption and utilisation, e.g. Zarkin et al. [22], Rodriguez Artalejo et al. [23] and Rice et al. [24]. Other studies have failed to find significant differences between abstainers and low-risk drinkers,, after controlling for rudimentary and health-related confounders [25,26]. Although this study employs a different outcome measure, i.e. alcohol-related diseases, the result seem to give some support to those studies that fail to find a protective effect on health care utilisation from low alcohol consumption compared to abstainers. Based on the epidemiological literature, one would expect an increase in health care utilisation from low alcohol consumption.

As discussed above, the result of the current study seems to sever the health link commonly used in the alcohol-wage literature, and the beneficial effects are found most pronounced for age-groups outside the labour force. The question that remains is how the 'missing link' between low alcohol consumption and increased wage should be explained. A number of other factors have been suggested such as family background and genetic endowment [4]. Human capital accumulation [27] has also been suggested, for example if alcohol consumption affects educational attainment. A third discussed issue is social networking [2] where it is often suggested that low consumers, as opposed to abstainers, join in after-work activities to a larger extent and thus creates a stronger social network that, for example, facilitates promotion. Another type of argument why low alcohol consumers tend to have the highest wages is that low alcohol consumption might increase *subjective *as opposed to *objective *(e.g. as measured in the current study) health. This possible advantage in subjective health might manifest itself in different manners such as improved quality-of-life, and/or reducing the number of days absent from work. Especially the latter is interesting as sick leave should affect wages both in short and long term. However, performed studies on the effect of alcohol consumption on sickness absenteeism show differentiated results [e.g. [28-30]]. Further studies are needed focusing on the link between low alcohol consumption and wages as there are many possible explanations and the literature in many related fields (e.g. alcohol and subjective health and sickness absence) is inconclusive [28-33].

It should be noted, however, that if diseases where low alcohol consumption has a protective effect are more important in terms of sickness absence (and other labour market outcomes) compared to those diseases with a detrimental effect of low alcohol consumption, the health link might still be valid. This would be in despite of the fact that most of the protective effects are for the retired population. This should be investigated in future research in addition to studies investigating if there are differences in effects between working and non-working individuals.

As is evident from the division of cost on inpatient-, outpatient and primary care, the net detrimental effect of low consumption is driven by the two later as inpatient care shows a protective effect. It has to be acknowledged that the data material for outpatient- and primary care are of a lower quality than inpatient care. If there is a serious bias in the reporting of disease codes in outpatient and primary care, for example if alcohol related diseases are given a disease code to a lower extent than non-alcohol related diseases, this could have a major effect on the results of this study. However, we expect such bias in coding to be more associated with diseases that are socially stigmatised, in this case fully alcohol related diseases such as alcohol abuse and alcohol dependence syndrome. Therefore are possible bias in outpatient and primary care of lesser concern as this type of diseases are excluded from the study due to the difficulties of dividing these on consumption groups. However, exclusion of fully alcohol related diseases and accidents have lead to an underestimation of the negative effect of low consumption. The assumption made in this paper was that the excluded diseases and accidents does not burden low alcohol consumption. The underestimation caused by this is perhaps most evident for injuries resulting from accidents, for example falls and fires caused by alcohol consumption, but also the risk of being victim of violence and accidents caused by others' consumption, where it seems probable that this could happen also to low consumers. This built-in underestimation can be expected to further strengthen the effect found in the study of increased medical care cost and episodes resulting from low alcohol consumption.

If the result of the current study holds and along with it the contradiction of the common link in the alcohol-wage literature, it implies that somewhere has a false step been taken. For example could the health link be invalid as an explanation of the wage premium from low alcohol consumption or, alternatively, could the underlying epidemiological literature be in error. The latter is by no means controversial as when research advances, new diseases are shown to have a medical relationship to alcohol consumption and current relationships are adjusted. The possibility of this, however, does not 'save' the health link as it should be based on the existing level of knowledge. Further studies are required, also using econometric methods, before any final conclusions can be made.

## Conclusion

The health link brought forward as an explanation in the alcohol-wage literature is based on *selected *information from the epidemiological literature. However, when conducting a study based on the *full *epidemiological information, i.e. inclusion of all alcohol-related diseases, it turns out that low alcohol consumption has a net detrimental effect. The health link therefore appears invalid and the result of the study is a discouragement of using health based on the epidemiological literature as a simplified and a not very well-reasoned link. An important drawback of using health as a default explanation is that other important factors are overlooked. A number of suggestions of possible factors that could constitute the link are given above. It is not unlikely that the link actually is compiled of several different factors that together give a significant effect of low alcohol consumption on wage. It should come as no surprise that the nature of links such as this is normally complex and care should be taken not to oversimplify.

## Conflict of interests

The authors declare that they have no competing interests.

## Authors' contributions

JJ and UGG designed and initiated the study. KHS estimated the prevalence of alcohol consumption in Sweden. JJ carried out the effect calculations. All authors have participated in the write-up phase and have approved the final manuscript.
